# Similarity, does it necessary mean plagiarism? Stop intentional and exaggerated paraphrasing

**DOI:** 10.3126/nje.v11i4.41137

**Published:** 2021-12-31

**Authors:** Eslam Elsayed Ali Shohda

**Affiliations:** 1 Dr. Eslam Elsayed Ali Shohda, Al ahrar teaching hospital, Zagazig, Egypt

Sir,

According to the Longman Dictionary of Contemporary English, plagiarism is; “when someone uses another person’s words, ideas, or work and pretends they are their own”, or it is “an idea, phrase, or story that has been copied from another person’s work, without stating where it came from”[1]. In other words plagiarism can be defined as “using someone else’s words, ideas or results without attribution ”[2]. Other scholar definitions are similar. The World Association of Medical Editors (WAME) defined plagiarism as ‘‘the use of others published and unpublished ideas or words (or other intellectual property) without attribution or permission, and presenting them as new and original rather than derived from an existing source’’ [3].

It is obvious that presenting others words, ideas or work with proper citation is not plagiarism. So similarity score whatever it is, is not necessary plagiarism. You may have a similarity score of 40% with a proper citation which means no plagiarism. At the same time, you may have a similarity score of 10% but expert reviewer detects a plagiarism. It is common now among researchers and many scientific communities that you should paraphrase your quotations to decrease similarity score whether manually or by using some programs to be blameless of plagiarism, which means that there is a confusion between both terms.

**Intentional paraphrasing**: is it a reasonable procedure or unnecessary time wasting?

It isn’t a reasonable procedure; it is unnecessary time wasting and it may lead to distortion of terminology or others ideas. Accumulation of knowledge can’t be denied, we use international scientific terms that shouldn’t be changed unless new concepts are formed (e.g. metabolism, quadriceps muscle, visual analog scale, law of attraction ….), even definition of terms is accepted universally and it is illogical to rephrase it. Paraphrasing complete sentences of previous researchers may change its meaning and this make these researchers carry what they did not exactly say, as those researchers might use the best words which expresses the intended meaning. In case of citing paraphrased sentences or ideas with much distortion from original work, this may delude readers that these are acceptable idea or facts meanwhile, in case of not citing these ideas or sentences this means that you impersonate or steal others' ideas.

Wallwork, 2011 stated that “Putting quotation marks (“...”) around an unaltered sentence and giving the proper citation for the origin of the work does not technically constitute plagiarism. But it may indicate to supervisors and referees that you have not actually understood what you have written – it is not your own work ”[4]. Using the original sentence in an unaltered form does not necessary mean that the researcher did not understand the meaning, sometimes the sentence is formulated in a clear form, sometimes it is a famous sentence in the scientific community and it may be results or a conclusion of previous work and the researcher found that it is better to put them unaltered.

If you want to paraphrase a sentence or a paragraph, you may find after this paraphrasing that your newly formed sentence is similar to some extent to another researcher who paraphrased the same sentence or paragraph, so we need an infinite words and this is illogical, many students and researchers complained much from this difficulty and time wasting. Even in arts and poetry, we transmit the poetic verses without changing their words while attributing them to those who said them. And we do not see a difference between this and scientific writing.

Meta-analysis, systemic reviews, review articles or critical appraisal studies are based on presenting previous results and conclusions, then analyzing them. So basically this will require a high similarity score.

## Do we need to repeat previous researches?

In many cases we need to repeat previous works or trials for several reasons:

- Previous researches may be restricted for many reasons to a specific sample of patients (women, diabetics, hospitalized patients, European descent…...) e.g. (Adherence to a low-risk, healthy lifestyle and risk of sudden cardiac death among women) we may need to know if previous results are the same among men. This is reasonable as restricting a research work to a specific sample of patients limits generalizability.
- Insufficient sample size which leads to insufficient statistical power to detect meaningful effects and this may produce unreliable results.
- We may need to repeat the same trial with some changes to the independent variable (modify dose, time, procedure….) e.g. (Effects of pulsed therapeutic ultrasound on the treatment of people with knee osteoarthritis) we want to know the effects of non-pulsed therapeutic ultrasound on the treatment of people with knee osteoarthritis.

In these previous cases and with proper citations there is no need to intentionally paraphrase the titles to escape from similarity, unless these changes are needed for the conduct of the study.

## Benefits of plagiarism programs for researchers

Researcher can review accuracy of quotations and citations. The researcher may find his own sentence or ideas are resembling other authors unintentionally, so he could put proper citation to avoid plagiarism or he could use this citation to support his idea.

## Benefits of plagiarism programs for reviewers

The reviewer can check if the researcher paraphrase sentences in a correct way that does not change the original meaning. If the researcher claims a new idea, the reviewer can know to what extent that is right.

Dr. Elsayed Ali Shohda Eslam, Al ahrar teaching hospital, Zagazig, Egypt

Dated the 24 December 2021

## References

[1] Longman Dictionary of Contemporary English [Internet]. United Kingdom: Pearson Education Limited; c 2018-2021 [cited 2021 Dec 23] Available from: https://www.ldoceonline.com/dictionary/plagiarism

[2] Cicutto L. Plagiarism: avoiding the peril in scientific writing. Chest. 2008;133(2):579-81. https://doi.org/10.1378/chest.07-2326 10.1378/chest.07-2326 PMid: 18252927

[3] World Association of Medical Editors [Internet]. Italy: Association; c 1995-2021 [cited 2021 Dec 23] Available from http://www.wame.org/resources/publication-ethics-policies-for-medical journals,2021

[4] Wallwork A. English for Writing Research Papers. New York: Springer;2011. p.154-155. https://doi.org/10.1007/978-1-4419-7922-3 10.1007/978-1-4419-7922-3 PMCid:

